# Synthesis, Characterisation and Antibacterial Properties of Silicone–Silver Thin Film for the Potential of Medical Device Applications

**DOI:** 10.3390/polym13213822

**Published:** 2021-11-05

**Authors:** Muhammad Faiz Aizamddin, Mohd Muzamir Mahat, Zaidah Zainal Ariffin, Irwan Samsudin, Muhammad Syafiek Mohd Razali, Muhammad ‘Abid Amir

**Affiliations:** 1 School of Physics and Material Studies, Faculty of Applied Sciences, Universiti Teknologi MARA, Shah Alam 40450, Malaysia; faizaizamddin@gmail.com; 2 School of Biology, Faculty of Applied Sciences, Universiti Teknologi MARA, Shah Alam 40450, Malaysia; drzaidah@uitm.edu.my; 3 Department of Cardiovascular and Thoracic Surgery, Faculty of Medicine, Universiti Teknologi MARA, Sungai Buloh Campus, Sungai Buloh 47000, Malaysia; irwansamsudin@uitm.edu.my (I.S.); syafiekrazali@uitm.edu.my (M.S.M.R.)

**Keywords:** silver particles, green synthesis, silicone film, conductivity, crystalline, antibacterial, medical application

## Abstract

Silver (Ag) particles have sparked considerable interest in industry and academia, particularly for health and medical applications. Here, we present the “green” and simple synthesis of an Ag particle-based silicone (Si) thin film for medical device applications. Drop-casting and peel-off techniques were used to create an Si thin film containing 10–50% (*v*/*v*) of Ag particles. Electro impedance spectroscopy (EIS), X-ray diffraction analysis (XRD), scanning electron microscopy (SEM), energy dispersive X-ray (EDX), and tensile tests were used to demonstrate the electrical conductivity, crystallinity, morphology-elemental, and mechanical properties, respectively. The oriented crystalline structure and excellent electronic migration explained the highest conductivity value (1.40 × 10^−^^5^ S cm^−^^1^) of the 50% Ag–Si thin film. The findings regarding the evolution of the conductive network were supported by the diameter and distribution of Ag particles in the Si film. However, the larger size of the Ag particles in the Si film resulted in a lower tensile stress of 68.23% and an elongation rate of 68.25% compared to the pristine Si film. The antibacterial activity of the Ag–Si film against methicillin-resistant *Staphylococcus aureus* (MRSA), *Bacillus cereus* (*B. cereus*), *Klebsiella pneumoniae* (*K. pneumoniae*), and *Pseudomonas aeruginosa* (*P. aeruginosa*) was investigated. These findings support Si–Ag thin films’ ability to avoid infection in any medical device application.

## 1. Introduction

Silver particles (Ag) or silver ions (Ag^+^) have been used as agents to combat bacterial infection through the incorporation of Ag into hundreds of healthcare and medical products [1,2,3,4]. The development of antibiotics has led to a thorough understanding of the efficacy and safety of these agents, which consume a high amount of time and resources. Concomitantly, infections caused by multi-resistant microorganisms keep growing and causing deaths worldwide [5,6]. Therefore, Ag has arisen as an excellent alternative because it can be applied to prevent infections caused by bacteria, decontaminate medical supplies, and even tackle infections in course. Properties such as low cytotoxicity and stability in human immunological response make Ag well-suited for medical applications such as coating, catheters, medicine, cosmetics, and dental implants [7,8,9,10,11]. Moreover, Ag was found to occupy a prominent place in human personal care products such as razors, contact lenses, and textiles while remaining compatible with any combination of substrates [12].

The potential of Ag as an antibacterial agent is related to its mechanisms of action, which attack microorganisms in multiple structures at a time and give them the ability to kill various types of bacteria. Pharmacologically, the antibacterial activity of synthesised Ag nanoparticles could be explained by several factors: (i) the destabilization of the outer bacterial membrane, (ii) the blocking of bacterial respiration, and (iii) the depletion of intracellular ATP leading to the denaturation of the bacterial cell wall [13,14,15,16,17]. A study by Yu et al. [18] showed that the synergistic effect of Ag and graphene oxide possessed prominent bactericidal capacities of 98.6% and 96.5% towards *Escherichia coli* and *Staphylococcus aureus*, respectively. Moreover, Ag particles promote wound contraction by differentiating fibroblasts into myofibroblasts, thereby increasing wound healing efficacy. In promoting dermal contraction and epidermal reepithelialisation during wound healing, Ag has been found to contribute to increased rates of wound closure [19,20].

Ag particles with high surface areas (nanoscale size below 100 nm) are of prime interest in exhibiting high antimicrobial actions against both Gram-positive and Gram-negative bacteria such as *Pseudomonas aeruginosa* and methicillin-resistant *Staphylococcus aureus* [4,21,22]. The selection of solvent and reducing agents should consider the reduction of metal salt to nanoparticles. Due to its biocidal actions, several physical and chemical routes have been applied to synthesize Ag [23,24,25,26]. The use of toxic precursors including sodium borohydride, potassium bitartrate, and hydrazine could be harmful to human tissue or skin [27,28,29]. As such, a tremendous amount of studies have shown that bio-synthetic plant extracts such as leaves are able reduce the oxidation state of Ag particles [30,31,32,33]. These biosynthetic synthesis processes remain challenging because they require long-term extraction and limit the imperfections in the particle structure of the product. Therefore, this study presents green-synthesis through the utilisation of an organic solvent without any extra additives and stabilisers as an alternative way to replace current procedures.

The growing interest in Ag particles warrants a biocompatible polymer coating substrate such as silicone (Si), polyvinylidene fluoride (PVDF), or chitosan [34,35]. Among them, Si offers versatile properties such as flexibility, transparency, flame resistance, and the capability of being formulated into various forms including gels, adhesives, and film coatings [36,37,38]. The application of Ag onto Si film opens a window for medical devices including body parts, catheters, shunts, and aesthetic implants [39]. Their fabrication process includes casting, peeling-off, printing, and spin-coating, which have been acknowledged allowing for controllable thicknesses that make the film formation and deposition of Ag more precise, reliable, and reproducible [40,41,42,43]. Although the combination of those materials is promising, the issues of particle deposition, size distribution, and mechanical performance still lack explanation. Based on this information, the present study describes the synthesis of Ag particles and the fabrication of silicone–silver (Si–Ag) thin films, wherein different key analyses were performed to characterise their crystalline, morphological, conductivity, and mechanical properties.

Nowadays, nosocomial bacterial infections, acquired from medical device formation and long-term use, have become widespread. Invasive devices such as catheters and ventilators employed under medical care in hospitals are often associated with these infections [44,45,46], which can become more dangerous when accompanied by considerably unpleasant symptoms such as fever that can lead to death [47,48]. Other studies [49,50,51] have reported that common nosocomial infectious bacteria such as methicillin-resistant *Staphylococcus aureus* (MRSA), *Bacillus cereus* (*B. cereus*), *Klebsiella pneumoniae* (*K. pneumoniae*) and *Pseudomonas aeruginosa* (*P. aeruginosa*) are initiated by the environment and dirty places, which increase the risk factors of hospital-acquired infections. To address these issues, intensive efforts have been devoted to design and fabricate antibacterial devices that incorporate antibacterial agents, such as antibiotics, cationic peptides, quaternary ammonium salts, metal nanostructures, and metal oxides, to prevent microbial growth [52]. The use of Ag-embedded Si films for medical devices could potentially reduce the frequency of such infections and substantially decrease morbidity and mortality. The higher affinity of Ag anchored to bacterial cell walls could cause physical changes in bacterial membranes, such as membrane damage, which could lead to cellular content leakage and bacterial death [53]. Therefore, we report here on the significant antibacterial properties of synergistic Si–Ag films against the abovementioned bacteria present in medical device applications.

## 2. Materials and Methods

### 2.1. Materials

Silver nitrate (AgNO_3_) (Mw = 169.87 g/mol) was purchased from Bendosen (Johor Bharu, Malaysia), dimethyl sulfoxide (DMSO) (ACS reagent, >99.9%) was supplied by Sigma Aldrich (St. Louis, MO, USA), and highly transparent LSR medical-grade liquid silicone gel rubber and its catalyst (acetoxy tin) were acquired from Foshan Tian Bao Li Silicon Engineering Technology Co., Ltd. (Guangdong, China).

### 2.2. Synthesis of Ag Particles

**Figure 1:**
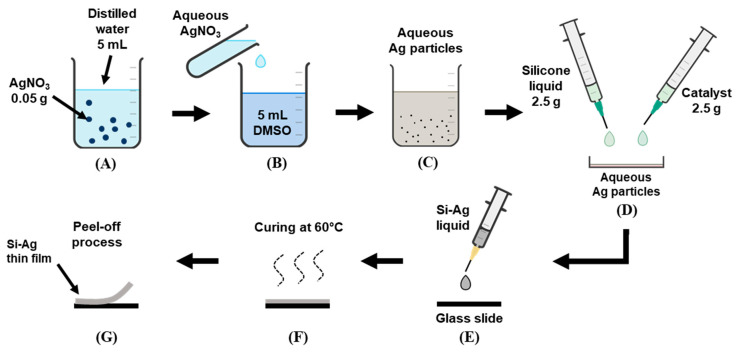
Illustration of the procedure for (**A**–**C**) the synthesis of Ag particles and the fabrication of Si–Ag thin films. (**D**) Si liquid and catalyst were mixed with aqueous Ag particles and (**E**) dropped onto the glass slide. (**F**) The curing process of Si–Ag liquid in oven and (**G**) peeled off of the fabricated film.

Briefly, 0.05 g of AgNO_3_ was diluted with 5 mL of distilled water and stirred for 30 min. Next, the aqueous AgNO_3_ was mixed with DMSO (reducing agent) at a 1:1 volume ratio (*v*/*v*) to initiate the formation of Ag particles, followed by a 4 h stirring process. The formation of Ag particles was noted completed after a greyish orange colour was physically observed. Before fabricating the film, the Ag particle solution was prepared in various concentrations from 10 to 50 volume percentage (*v*/*v*). The graphical procedure is illustrated in Figure 1A–C.

### 2.3. Fabrication of Si–Ag Thin Films

A 1:1 liquid ratio (2.5 g) of medical-grade Si liquid and catalyst was carefully mixed into the 10% aqueous Ag particle solution. The catalyst was utilised to speed up the film formation reaction. Following this, the mixture (Si–Ag) liquid was dropped onto the glass slide and subsequently subjected to an oven for the curing process. To ensure a complete surface finish, the process was conducted for about 2 h at 60 °C. The Si–Ag thin film was peeled off after cooling at room temperature. The overall process was repeated for other particle solutions with different concentrations of Ag. Figure 1D–G illustrates the procedure of the abovementioned approach.

### 2.4. Characterisation of Si–Ag Thin Films

#### 2.4.1. Film Thickness Measurement

Film thickness was measured with a high-precision digital 0–25 mm screw gauge (Foshan Songqi Technology Co., Ltd., Foshan, China). The average reading was recorded after five measurements were taken at different film surfaces.

#### 2.4.2. Observation of Morphology, Elemental Composition, and Ag Diameter

The surface morphology and elemental composition of the Si–Ag thin films were investigated with scanning electron microscopy (SEM) (SNE-4500M Plus Tabletop SEM, SEC Co., Ltd., Suwon-si, Korea) and energy dispersive X-ray (EDX) analysis, respectively. Prior to this, the films were subjected to gold-sputtering to enhance the image resolution. Next, the diameter of the Ag particles was measured with ImageJ software (Version 1.53k, National Institutes of Health, New York, NY, USA) [54,55,56].

#### 2.4.3. Electrical Conductivity Measurement

The electrical conductivity of the Si–Ag thin films was measured with electro impedance spectroscopy (EIS) (HIOKI 3532-50 LCR-HI Tester, HIOKI E. E. Corporation, Nagano, Japan). The analysis was conducted at room temperature with a frequency range from 100 to 1000 kHz. The film (3 × 3 cm^2^) was placed between two copper electrodes with a 1 cm diameter. The measurements were taken three times, and the average reading was recorded. Conductivity measurements were derived from the following expression [57,58,59,60,61]:

$$\sigma =\frac{L}{\mathrm{Rb}\times A}$$

where σ is conductivity, Rb is the bulk resistance measured by the instrument, L is the film thickness, and A is the cross-sectional area of the electrode.

#### 2.4.4. Mechanical Characteristics

The tensile properties of the Si–Ag thin films were determined with a universal strength tester machine (Tenso Lab 5000, MESDAN SPA, Brescia, Italy). The rate of transverse and load force of each sample were 300 mm/min and 5 kN, respectively. Tensile stress (N) and elongation (%) were recorded in the analysis.

### 2.5. Antibacterial Function

A Kirby–Bauer disc diffusion test was performed to investigate the antibacterial properties of the Si–Ag thin films. The films were subjected to Gram-positive bacteria, methicillin-resistant *Staphylococcus aureus* (MRSA) and *Bacillus cereus* (*B. cereus*), and Gram-negative bacteria, *Klebsiella pneumoniae (K. pneumoniae)* and *Pseudomonas aeruginosa* (*P. aeruginosa*). Prior to this, the bacterial growth strains were inoculated with 4.0 × 10^−7^ CFU mL^−1^ and incubated at 37 °C for 24 h. With sterilised tweezers, the specimens were placed onto an agar layer including the positive control antibiotic, pristine Si thin film, and 50% Ag–Si thin film. The specimens were incubated in Petri dishes for 24 h at 37 °C. The appearance and diameter of the inhibition zones around the specimens were recorded. Measurements were performed in triplicate.

## 3. Results and Discussion

### 3.1. Fabrication of Si–Ag Thin Films

**Figure 2:**
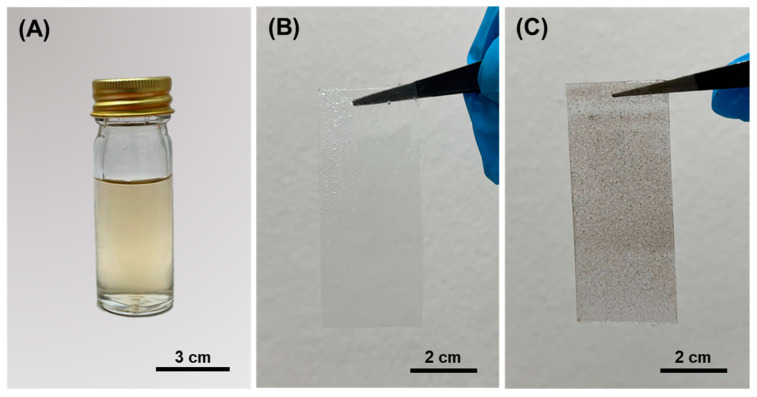
(**A**) Aqueous Ag particles from AgNO_3_ with the addition of DMSO; the physical appearance of (**B**) pristine Si film and (**C**) Si–Ag thin film.

The Ag particles were synthesised from AgNO_3_ in a DMSO solvent by reducing Ag^+^ into Ag^0^ atoms. Our study showed that Ag particles could be produced when subjected to the DMSO solvent as the reducing agent at room temperature (pH 6–7) without any additional additives. The colour changed from transparent to greyish orange, indicating that Ag particles were formed [15,62,63] (Figure 2A). Notably, we can unequivocally state that the synthesis of Ag particles occurred via a mechanism that was distinct from other established methods. This was because we used no extra additives and stabilisers while the reaction was conducted at room temperature. In comparison to other studies [64,65,66,67], our approach could possibly lead to an inconsistent synthesis reaction. According to the nucleation theory on synthesising nanoparticles, the nuclei (seeds) of Ag particles define the minimum size at which the particles can survive in solution without being redissolved. The same is true for the particles’ free energy, where critical free energy is required to obtain stable particles within solution [68]. The unstable reaction of grain formation could also lead to the broad and random size distribution of Ag particles. One may observe a low rate of Ag ion reduction without the proper temperature (>45 °C), which also indicates larger diameters of produced particle [69]. Nevertheless, we synthesised Ag particles in a DMSO solvent in which DMSO was the most important reducing agent at room temperature and in neutral pH conditions. Although the reaction could be inconsistent, the Ag particles were still produced. We also considered that this method could be seen as a “green” synthesis method due to its reliable, simple and eco-friendly protocol for synthesising metal oxides such as Ag.

The fabrication of Si–Ag films was achieved through drop-casting due to its simplicity, precise thickness formation, and easy film removal via peeling from the substrate [70]. Five concentrations of Ag particles (10, 20, 30, 40 and 50%) were prepared and mixed with Si liquid. We added the Si catalyst to the mixture to accelerate the reaction. Meanwhile, the pristine Si film was considered the control variable (absent of any Ag particles). The physical appearances of the pristine Si film and the Si–Ag film are displayed in Figure 2B,C, respectively. The distribution of greyish colour in the Si–Ag film distinguished the Ag particles that were successfully fabricated on the surface of the Si film. The average thickness value of each film is reported in Table 1.

Drop-casting is an ideal procedure used to obtain a film consisting of coherent domains [71]. We observed that there was a 1-fold increase in thickness upon the addition of Ag particles. The modification of thickness may have been affected by the diameter and distribution of Ag particles in the Si film [72]. Moreover, the micrometre (mm) scale thickness could have been caused by the aggregation of silver particles and trapped bubbles during the fabrication process, which facilitated uneven surface structures and large internal voids, respectively.

### 3.2. Electrical Conductivity of Si–Ag Thin Films

**Figure 3:**
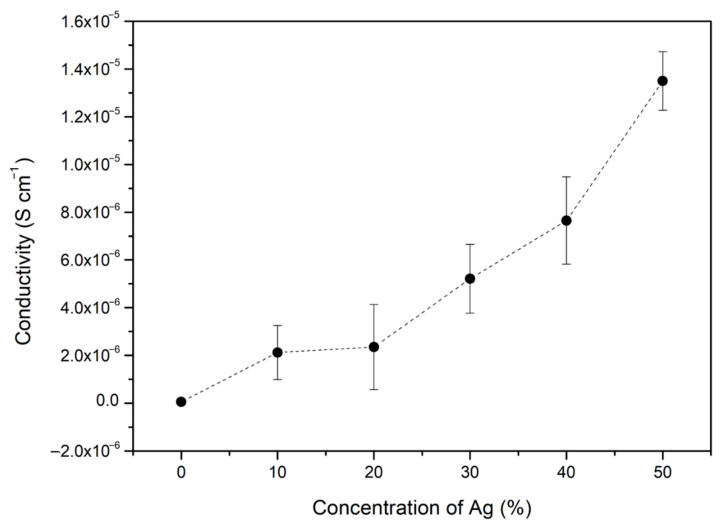
Trends in the conductivity of Si–Ag thin films with the addition of Ag. The error bar indicates the uncertainty of a reported measurement.

EIS measurements were carried out to verify the electrical conductivity of the Si–Ag thin films under different concentrations of Ag (Table 2). Pristine Si film exhibited insulation due to the lack of free electrons moving in the film [73]. Conductivity was initiated by the addition of 10% Ag to the Si film, which exhibited electrical behaviour with a conductivity value of 2.05 × 10^−6^ S cm^−1^. The separation between Ag particles by the Si matrix was significantly larger at low metal contents than high metal contents. This condition disrupted the diffusion and migration range of electrons [72]. As the concentration of Ag was increased, the measured conductivity rapidly increased. The 50% Ag–Si film achieved the highest conductivity value of 1.40 × 10^−5^ S cm^−1^. The trend of conductivity and Ag addition is plotted in Figure 3. This increment was possibly caused by the interconnectivity and aggregation of Ag particles formed in the Si film. The cohesion and contact promoted a large surface area, which induced charge migration. Moreover, the increment of 1 magnitude originated from the formation of an enlarged metallic pathway. The 50% concentration of Ag provided the highest composition of metallic particles, thus allowing the particles to directly contact each other frequently. This action suggests the complete agreement of metallic network forms along with the polymer matrix. Without the matrix cover and a large percentage of Ag (metal-to-metal contact), an efficient electrical charge could be produced, though this conduction mechanism was dependent on the crystallinity and size of the Ag particles [74,75].

### 3.3. Crystallinity-Structural Study

**Figure 4:**
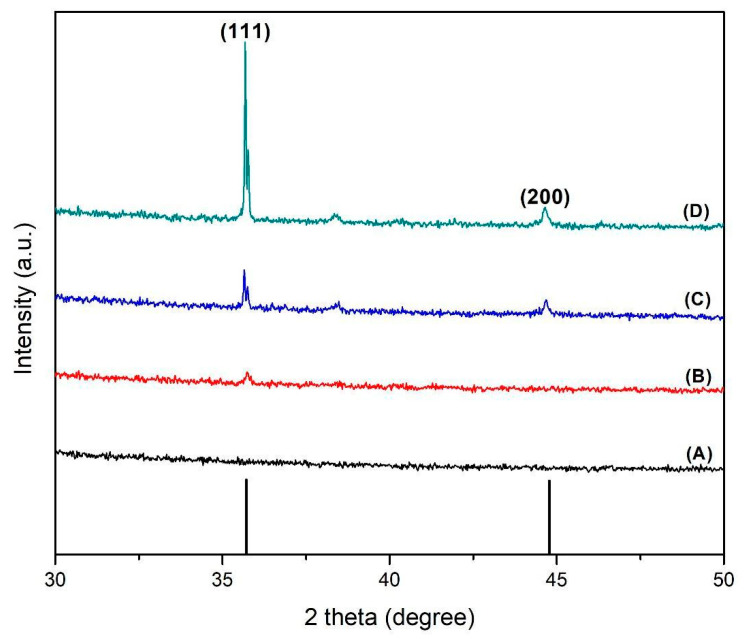
X-ray diffraction patterns of (**A**) pristine Si film, (**B**) 30% Ag–Si film, (**C**) 40% Ag–Si film, and (**D**) 50% Ag–Si film.

The crystalline structures of the Si–Ag thin films were assessed with XRD analysis. Figure 4 displays the peak characteristics observed at angle 2 theta (from 30° to 50°), which confirmed the presence of the Ag polycrystalline phase. The intensity was directly proportional to the additional concentration of Ag particles. The peaks at angles of 36° and 44.5° corresponded to the polycrystalline Ag particles on the Si films, which were explained by cubical structures of Ag phase directions (111) and (200), respectively [76,77]. The diffraction peaks were recorded for the face-centred cubic (FCC) structure, in accordance with other past research [78,79]. Moreover, the highest peak intensity demonstrated by the 50% Ag–Si film (Figure 4D) was attributed to the lowest surface energy provided by the Ag particles. In other words, the excellent degree of crystallinity, associated with the intermolecular forces between Ag grains, provided the lowest adhesion to the Si substrate [80]. Nevertheless, the sharp peaks and highest intensity patterns suggested that Ag particles were crystalline and highly oriented in this experiment.

### 3.4. Morphological Analysis of Si–Ag Thin Films

**Figure 5:**
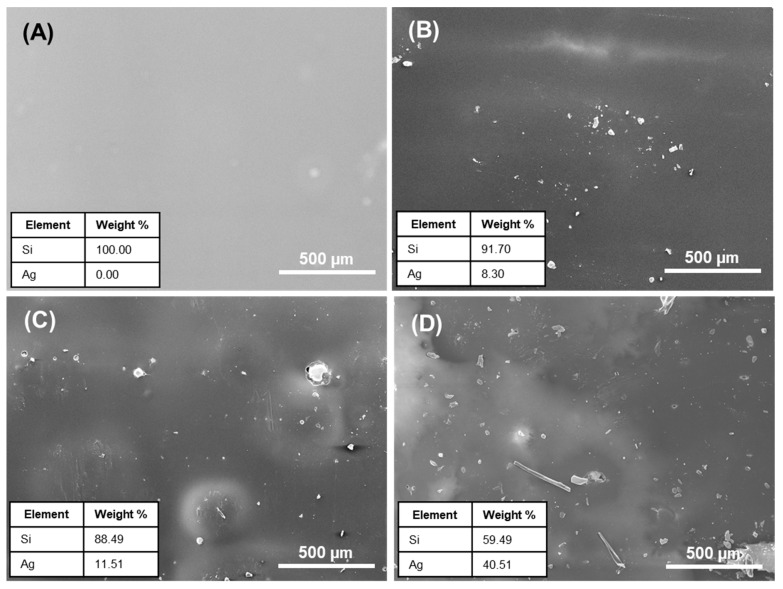
Morphological images and elemental analysis of (**A**) pristine Si thin film and (**B**) 30% Ag–Si, (**C**) 40% Ag–Si, and (**D**) 50% Ag–Si thin films.

The morphological properties of the Si–Ag thin films were closely linked to the crystallinity and electrical properties of the Ag particles. Factors such as the size and crystallinity of Ag particles could lead to excellent electrical conduction [81]. Here, we performed SEM and EDX analyses to determine the morphological and elemental composition of the Si–Ag thin films, respectively. Figure 5 shows micrograph images of pristine Si and Si–Ag thin films. The pristine Si thin film displayed a homogenous surface with the absence of Ag particles (Figure 5A). Moreover, we observed that some microstructures on the film, which could be attributed to the trapped microstructure bubbles. This microstructure was responsive to gas diffusions such as nitrogen (N) and oxygen (O) from surrounding voids. Godbole et al. [82] asserted that O gas is easily attracted to the surface of Si, and this attraction can be utilised via air during film formation. The trapped bubbles may change the overall effective properties of a film, such as conductivity and mechanical behaviour. In conductivity measurements, a significant number of bubbles could produce weaker conductance and add to disorderly film thickness [83].

**Figure 6:**
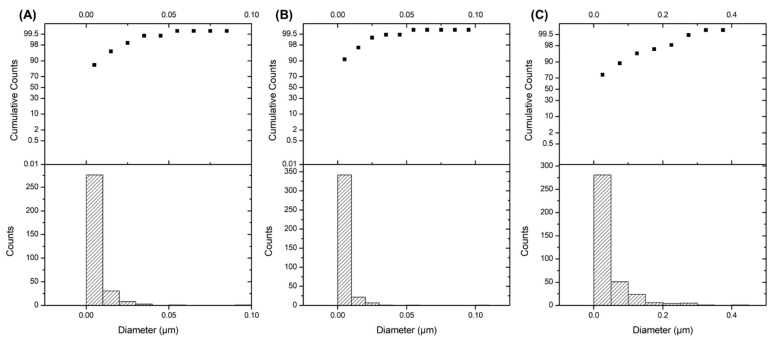
Diameter and distribution of (**A**) 30% Ag, (**B**) 40% Ag and (**C**) 50% Ag particles on the Si films, as analysed via ImageJ software.

Figure 5B shows the distribution of the 30% Ag particles (confirmed by EDX) on the surface of the Si films. The image shows a random distribution of Ag particles with an approximately 0.0054 µm diameter. Surprisingly, different geometric shapes of Ag particles, such as polyhedron and cylinder-like, adhered to the Si film (Figure 5C,D). This action was possibly due to the agglomeration and cohesion of grains during the curing process [84]. A change in chemical reaction and interconnection between the Ag grains promoted the tightly packed and oversized grain particles. The obtained images were analysed with the ImageJ software to determine the size, diameter, and distribution of the Ag particles on the Si films (Figure 6). The average diameter of Ag particles is tabulated in Table 3. The Ag particle diameter was significantly larger in the 50% Ag–Si film, followed by the 40% and 30% Ag–Si films. Different and increased grain sizes led to the enrichment of crystallite quality and the reduction of grain boundary scattering in the films [85]. Moreover, these size differences significantly reduced interparticle spacing and increased electron tunnelling mobility. In other words, the Ag particles created an electrically conductive network.

### 3.5. Mechanical Properties of Si–Ag Thin Films

**Figure 7:**
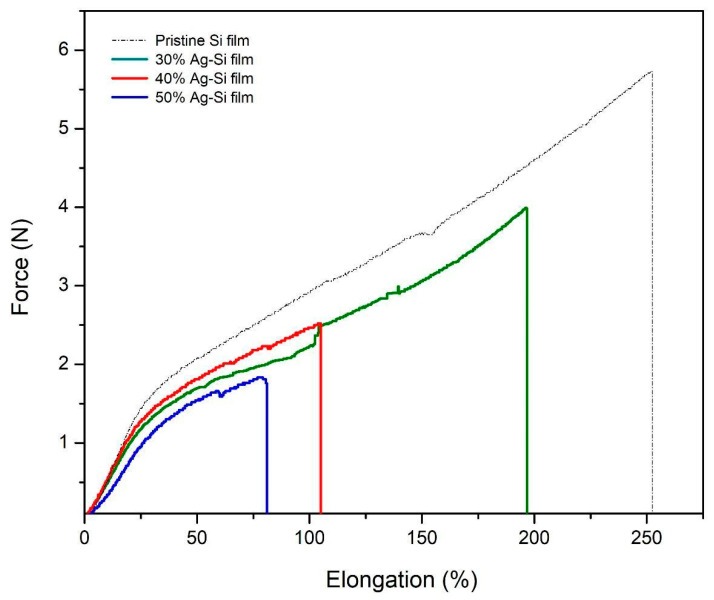
Tensile stress and elongation of the Si–Ag thin films as a function of different Ag compositions.

Thin films should be provided excellent mechanical stability to ensure their durability. The tensile stress (N) and elongation rate (%) of the Si–Ag thin films as a function of Ag composition (30%, 40% and 50%) were evaluated with tensile tests; the results are presented in Figure 7. The pristine Si film displayed the highest tensile stress and elongation rate values of 5.73 N and 252.14%, respectively. The trend showed a decrement for both tensile stress and elongation upon the addition of Ag particles onto the Si film. All the data are reported in Table 4.

In the 30% Ag–Si film, the tensile stress and elongation showed slight decreases of 30.89% and 22.23%, respectively, compared to the initial pristine Si film. The addition of Ag particles significantly hampered the mechanical properties of the film. As mentioned in the previous section, crystallinity plays a role in the structure and morphology of Si–Ag films. Compared to the control sample, the 40% and 50% Ag–Si films presented reduced tensile stresses of 56.02% and 68.23%, respectively, and elongation rates of 58.65% and 68.25%, respectively. Inconsistent but larger grain sizes could be produced by increasing the concentration of Ag particles. According to the tensile test, crystallite grains or interconnection between the grains could play a major role in the tensile properties [86,87]. Although crystalline materials are stiffer due to grain-boundary-related hardening, other factors such as larger-sized of grains could lead to greater collisions between particles and consequently the breaking point [88]. Moreover, Kong et al. [89] asserted that the stress-concentration phenomena of Si films caused by the lack of particle or matrix adhesion of Ag could lead to reductions of elongation and tensile stress. The bubbles or voids in the film produced during the fabrication led to the creation of cavities, which was also caused the reduction in the tensile stress of the Si–Ag films. Under the tensile test, these voids added to micro-scale damage and evolved faster when combined with Ag particles [90]. Therefore, the materials were more easily deformed by weaker local stress.

### 3.6. Antibacterial Activities of Si–Ag Thin Films

**Figure 8:**
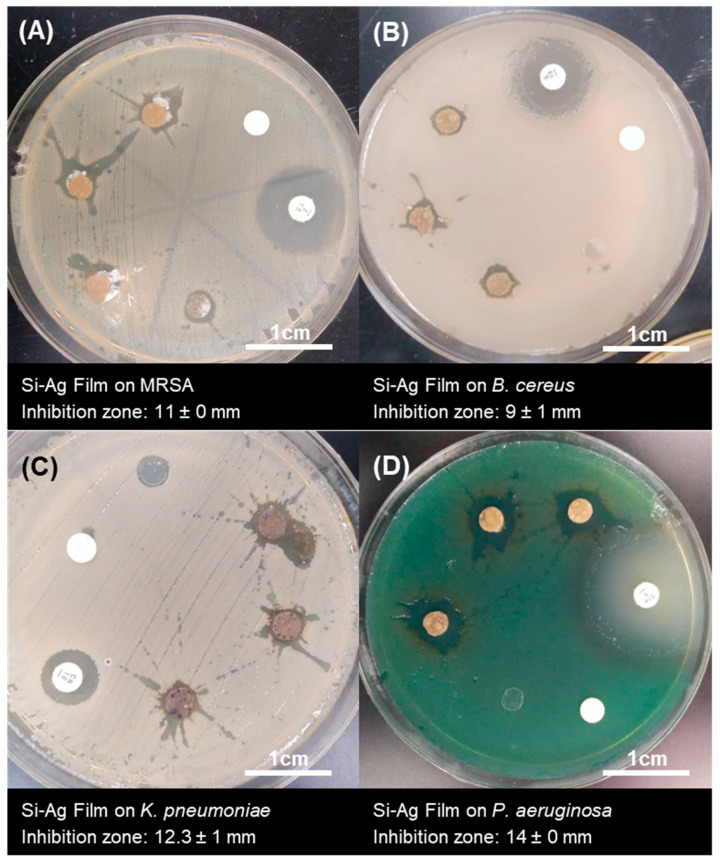
The inhibition zone of Si–Ag thin films against Gram-positive bacteria, (**A**) MRSA and (**B**) *B. cereus*, and Gram-negative bacteria, (**C**) *K. pneumoniae* and (**D**) *P. aeruginosa*, as evaluated with the Kirby–Bauer disk diffusion method. Measurements were performed in triplicate.

Silver ions (Ag^+^) or silver particles (Ag) can destroy multiple drug-resistant pathogens and distort their growth formation, thus imbuing them with significant potential in antibacterial applications [91]. Although the antibacterial mechanisms of Ag have been thoroughly discussed, the combination effect of Ag in Si film is still underexplored. The antibacterial activity of Si–Ag films was tested against MRSA and *B. cereus* as Gram-positive bacteria representatives and *K. pneumoniae* and *P. aeruginosa* as Gram-negative bacteria representatives (Figure 8). Antibacterial activity was demonstrated with the Kirby–Bauer disc diffusion method, which is described as a preliminary method for screening the antibacterial activity of antimicrobial agents [61,92]. Our results are summarised in Table 5. The presence of an inhibition zone around the Si–Ag films suggested that the films possessed antibacterial activity that was able to inhibit the growth of inherent pathogens. Weak inhibition zones of 11 ± 0 and 9 ± 1 mm, respectively, were displayed by the Gram-positive bacteria MRSA and *B. cereus*. On the contrary, the Gram-negative bacteria *K. pneumoniae* and *P. aeruginosa* presented inhibition zones of 12.3 ± 1 and 14 ± 0 mm, respectively. As mentioned above, the antibacterial effect of Si–Ag film on Gram-negative bacteria was found to be stronger than that on Gram-positive bacteria. This phenomenon can be explained by the differences in cell wall thickness between Gram-positive bacteria (20–80 nm) and Gram-negative bacteria (7–8 nm), which are mainly composed of peptidoglycan layers [93]. Therefore, Ag has a high affinity for anchoring onto and consequently infiltrating bacterial cell walls, which could cause physical changes in bacterial membranes, such as membrane damage, that can lead to cellular content leakage and bacterial death [53].

In general, the cellular membranes of bacteria are surrounded by negative charges (−) due to the presence of phosphate, carboxyl and amino groups [14]. These charges could facilitate electrostatic interactions between the positive charge (+) of Ag and cell membranes. This condition imparts antibacterial activities by altering the surface charge and applied attractive forces. Simultaneously, Ag can penetrate and enter the cells of bacteria, resulting in interactions with cellular structures such as proteins, lipids, and DNA [9]. Lok et al. [94] claimed that the oxidation state of metals such as copper (Cu) and Ag could contribute to their bactericidal effects. The phenomenon of O_2_ reacting with Ag_2_O and forming Ag^2+^ produces a superoxide (O^2−^) that regenerates H_2_O_2._ The process continuously reduces Ag^2+^ to Ag^+^ and increases the oxidative stress towards the bacterial membrane, leading to bacterial dysfunction and death. This denaturation condition was clearly seen by the inhibition zone formed around the layer of the film in this study. The Si–Ag thin films showed antibacterial activity against all tested bacteria. However, they were classified in the range of resistant susceptibility, which suggested weak antibacterial functions. We noted some major points that discouraged antibacterial activity. Based on the SEM micrographs (see Section 3.3) following the synthesis of Ag particles, we achieved particle sizes of approximately ~30–50 µm. According to Wang et al. [95], Ag particles with large diameters could interrupt potentials during interactions with bacterial membranes, therefore causing less penetration and limited antibacterial activities. Additionally, the distribution of Ag particles in the Si films played an important role in providing excellent antibacterial functions. Yin et al. [4] asserted that Ag nanoparticles can continually release silver ions, which may be involved in the mechanisms of bacteria death. Ag’s close-range and even distribution has the potential to maximize antibacterial activity. Finally, some concerns involving the charge diffusion barriers of Si films were also highlighted. Si substrates are well-utilised in medical applications due to their flexible and non-toxic properties. Nevertheless, the main antibacterial function of Ag may be an issue when incorporated into Si substrates. The electromigration of Ag in Si substrates differs, thus limiting antibacterial activity [96]. Moreover, the hydrophobic nature of Si could become dominant and lead to weak electrostatic interactions between Ag particles and bacteria. The change in the dominance of electrostatic and hydrophobic Si composite interactions might also provide an explanation for their effectiveness against bacteria [97].

## 4. Conclusions

In this study, Ag particles were successfully synthesised using a reducing agent (DMSO) and no additional additives (green synthesis). Next, the Ag particles were incorporated in the Si film using the drop-casting and peel-off techniques. Following the addition of Ag at various concentrations, the film thickness (from ~5.12 to 6.78 mm) was affected by the uneven size and distribution of the Ag particles. The experimental results revealed that the electrical conductivity was initially increased by the addition of 10% Ag. The 50% Ag–Si film had the highest conductivity value of 1.40 × 10^−5^ S cm^−1^, which could be attributed to Ag particle cohesion and resulted in larger grains that allowed electrons to flow along the Si matrix. The crystallinity of Ag particles was suggested to be one of the main reasons for their excellent conductivity values. The presence of crystalline structure and highly oriented Ag grains, according to XRD analysis, may aid in electronic migration. The SEM analysis revealed that the Ag particles had polyhedron and cylinder-like shapes, which supported the conductive network in the film. Despite this, the irregular shape of the Ag grains was identified as a disadvantage in the mechanical properties of the film, as it resulted in 68.23% and 68.25% lower tensile stress and elongation rates, respectively, compared to the pristine Si film. As the size of Ag increased, the collision between the particles became more intense and the film reached its breaking point. The antibacterial properties of the Ag and Si films were synergistic against MRSA, *B. cereus*, *K. pneumoniae*, and *P. aeruginosa*. This denaturation condition was clearly demonstrated by the inhibition zone formed around the layer of the film on the surface of the aforementioned bacteria. These findings may be used to hinder infections on the surfaces of medical device applications.

## Figures and Tables

**Table 1:** List of samples and their film thickness.

No.	Sample	Thickness (mm)
1.	Pristine Si film	5.12 ± 0.03
2.	10% Ag–Si film	5.38 ± 0.14
3.	20% Ag–Si film	5.83 ± 0.03
4.	30% Ag–Si film	6.21 ± 0.02
5.	40% Ag–Si film	6.34 ± 0.03
6.	50% Ag–Si film	6.78 ± 0.03

**Table 2:** Conductivity measurement of Si–Ag thin films.

No.	Concentration of Ag (%)	Conductivity (S cm^−1^)
1.	Pristine Si film	NIL
2.	10	2.05 × 10^−6^ ± 1.04 × 10^−6^
3.	20	2.43 × 10^−6^ ± 1.63 × 10^−6^
4.	30	5.03 × 10^−6^ ± 1.23 × 10^−6^
5.	40	7.52 × 10^−6^ ± 1.89 × 10^−6^
6.	50	1.40 × 10^−5^ ± 1.39 × 10^−4^

**Table 3:** The average Ag particle diameter for each sample.

No.	Sample	Average Diameter of Ag Particles (µm)
1.	Pristine Si film	NIL
2.	30% Ag–Si film	0.0054
3.	40% Ag–Si film	0.0046
4.	50% Ag–Si film	0.0656

**Table 4:** The tensile stress and elongation rate of each sample.

No.	Sample	Tensile Stress (N)	Elongation Rate (%)
1.	Pristine Si film	5.73	252.14
2.	30% Ag–Si film	3.96	195.92
3.	40% Ag–Si film	2.52	104.26
4.	50% Ag–Si film	1.82	80.06

**Table 5:** Measurements of inhibition zones of Ag–Si thin films in contact with bacteria.

Type of Bacteria	Antibiotic Disc	Inhibition Zone (mm)	Zone Diameter Breakpoints (mm)		
			Resistant	Intermediate	Susceptibility
MRSA	Clindamycin	11 ± 0	≤14	15–20	≥21
*B. cereus*	Streptomycin	9 ± 1	≤11	12–14	≥15
*K. pneumoniae*	Amikacin	12.3 ± 1	≤14	15–16	≥17
*P. aeruginosa*	Ciprofloxacin	14 ± 0	≤15	16–20	≥21

## Data Availability

Not applicable.
